# Diacidic Motifs in the Carboxyl Terminus Are Required for ER Exit and Translocation to the Plasma Membrane of NKCC2

**DOI:** 10.3390/ijms232112761

**Published:** 2022-10-23

**Authors:** Dalal Bakhos-Douaihy, Elie Seaayfan, Nadia Frachon, Sylvie Demaretz, Martin Kömhoff, Kamel Laghmani

**Affiliations:** 1 Centre de Recherche des Cordeliers, INSERM, Sorbonne Université, Université de Paris, F-75006 Paris, France; 2 CNRS-ERL8228, F-75006 Paris, France; 3 Division of Pediatric Nephrology and Transplantation, University Children’s Hospital, Philipps-University, 35043 Marburg, Germany

**Keywords:** kidney, Bartter syndrome, trafficking, ER export, COPII

## Abstract

Mutations in the apical Na-K-2Cl co-transporter, NKCC2, cause type I Bartter syndrome (BS1), a life-threatening kidney disease. We have previously demonstrated that the BS1 variant Y998X, which deprives NKCC2 from its highly conserved dileucine-like motifs, compromises co-transporter surface delivery through ER retention mechanisms. However, whether these hydrophobic motifs are sufficient for anterograde trafficking of NKCC2 remains to be determined. Interestingly, sequence analysis of NKCC2 C-terminus revealed the presence of consensus di-acidic (D/E-X-D/E) motifs, ^949^EEE^951^ and ^1019^DAELE^1023^, located upstream and downstream of BS1 mutation Y998X, respectively. Di-acidic codes are involved in ER export of proteins through interaction with COPII budding machinery. Importantly, whereas mutating ^949^EEE^951^ motif to ^949^AEA^951^ had no effect on NKCC2 processing, mutating ^1019^DAE^1021^ to ^1019^AAA^1021^ heavily impaired complex-glycosylation and cell surface expression of the cotransporter in HEK293 and OKP cells. Most importantly, triple mutation of D, E and E residues of ^1019^DAELE^1023^ to ^1019^AAALA^1023^ almost completely abolished NKCC2 complex-glycosylation, suggesting that this mutant failed to exit the ER. Cycloheximide chase analysis demonstrated that the absence of the terminally glycosylated form of ^1019^AAALA^1023^ was caused by defects in NKCC2 maturation. Accordingly, co-immunolocalization experiments revealed that ^1019^AAALA^1023^ was trapped in the ER. Finally, overexpression of a dominant negative mutant of Sar1-GTPase abolished NKCC2 maturation and cell surface expression, clearly indicating that NKCC2 export from the ER is COPII-dependent. Hence, our data indicate that in addition to the di-leucine like motifs, NKCC2 uses di-acidic exit codes for export from the ER through the COPII-dependent pathway. We propose that any naturally occurring mutation of NKCC2 interfering with this pathway could form the molecular basis of BS1.

## 1. Introduction

The importance of the thick ascending limb (TAL) of Henle’s loop in renal physiology and pathophysiology is evidenced by its critical roles in blood pressure homeostasis, water balance, acid-base regulation and divalent mineral cation metabolism [1,2]. The apically located Na-K-2Cl cotransporter, NKCC2, is the pacemaker of sodium chloride reabsorption in the TAL [2,3,4]. Consequently, NKCC2 transport function is indispensable for many tasks of this nephron segment. Inactivating NKCC2 mutations causes antenatal Bartter syndrome type-1, a life-threatening kidney disease which is diagnosed usually in the antenatal period, due to the presence of fetal polyuria leading to polyhydramnios and preterm labor, salt loss, hypokalemia, metabolic alkalosis, hypercalciuria, and nephrocalcinosis [5,6]. Without appropriate treatment, patients with BS1 do not survive, in general, the early neonatal period [6]. In line with the severity of the BS1 symptoms, we showed recently that all tested NKCC2 mutations impair cotransporter transport activity and cell surface expression. Moreover, we demonstrated that the ER retention and associated protein degradation (ERAD) seems to be the most common mechanism underpinning BS1 [7]. Hence, we provided evidence that Bartter syndrome type 1 is among diseases linked to the ERAD pathway. In this regard, it is conceivable that NKCC2 folding mutants do not pass ER quality control, leading to their retention in the ER. However, another possibility is that recognition of an ER exit sequence by components of the ER sorting machinery is impaired in NKCC2 mutants. In support of this notion, we previously demonstrated that any premature termination, such as BS1 mutant Y998X, interfering with highly conserved di-leucine like motifs of NKCC2 C-terminus, impairs the co-transporter surface delivery due to defective trafficking [8,9]. We therefore proposed abnormal trafficking as a common BS type I mechanism associated with inactivating mutations of NKCC2 deleting the distal region of the cotransporter C-terminal tail. Collectively, all these observations clearly highlight the fact that ER quality control and ER export represent critical steps in NKCC2 trafficking to the cell surface. Consequently, to develop therapeutic strategies aiming to promote NKCC2 mutants trafficking from the ER to the cell surface, it is crucial to understand the mechanisms and pathways that regulate their export from the ER.

The proper sorting of transmembrane proteins to the cell surface is governed by specific sorting signals, which are generally present in their cytoplasmic domains [10,11]. NKCC2 belongs to the super family of electroneutral cation-coupled chloride co-transporters that exhibit similar hydropathy profiles with 12 transmembrane spanning domains, an NH2 terminal tail of variable length, and a long cytoplasmic COOH-terminus. Given that the C-terminal domain of NKCC2 is the predominant cytoplasmic region, we postulated that it plays a major role in the cotransporter trafficking. In support of this idea, several reports demonstrated that COOH-terminal residues are essential for correct protein targeting [12,13,14]. One type of sorting signal important for protein export from the ER is mediated by so-called di-leucine-like motifs. [15,16,17]. In this regard, we previously reported that highly conserved di-leucine-like motifs present in the C-terminal tail of NKCC2 and the structurally related Na-Cl cotransporter NCC, are required for their ER exit and cell surface expression [8,9]. Besides these di-leucine/di-hydrophobic motifs, di-acidic motifs (D/E)*X*(D/E) also mediate the ER export of several transmembrane proteins such as CFTR and TASK-3 [18,19]. These di-acidic signals direct selective ER export by promoting interaction of cargo proteins with coat complex II (COPII) budding machinery [20]. Of note, while it was initially thought that COPII coat complex is absolutely required for ER exit, more recent reports revealed that there is an increasing number of proteins that leave the ER independently the COPII machinery [21,22,23]. In this regard, whether COPII plays a pivotal role in the ER exit of NKCC2 remains to be clarified. Herein, we reveal that COPII is essential for export of NKCC2 from the ER. Moreover, we show that the previously identified di-leucine motifs of NKCC2 are necessary, but not sufficient, to mediate the ER exit of the co-transporter. Indeed, we have now revealed the requirement of di-acidic motifs in the ER-to-Golgi trafficking of NKCC2. Loss of these motifs or COPII disrupts ER export and efficient transport of NKCC2 to the plasma membrane. Thus, our study provides new insights into the intracellular trafficking of NKCC2 by revealing a new regulatory mechanism governing the ER-to-Golgi transport of the cotransporter.

## 2. Results

### 2.1. Identification of a New Pure ER Exit Signal in NKCC2 COOH-Terminus

**Figure 1:**
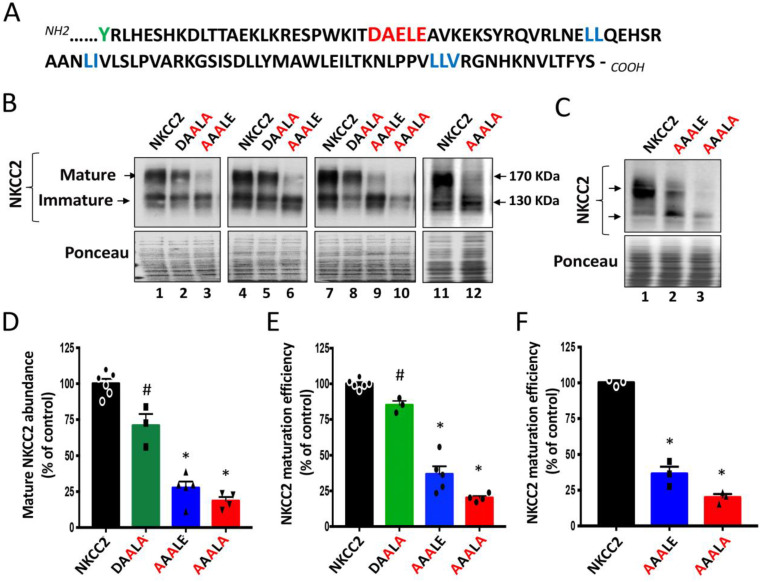
Identification of di-acidic ER export codes in NKCC2 COOH-terminal domain. (**A**) In addition to the previously identified di-leucine motifs (blue), the distal region of NKCC2 COOH terminus contains also a potential ER export signal, the DAELE motif (red), located downstream from Y residue (green), where nonsense mutation Y998X occurs. (**B**) Immunoblot analysis of OKP cells expressing wild-type or mutant NKCC2 proteins as indicated. Cells were transiently transfected with WT NKCC2 or mutated NKCC2 proteins; ^1019^AAALE^1023^ or ^1019^AAALA^1023^. Forty-eight hours post-transfection, total cell lysates were separated by SDS-PAGE and probed by anti-Myc antibodies. The positions of the core (immature) and complex-glycosylated (mature) proteins are indicated. The lower panel is the same membrane stained with Ponceau S to illustrate the equal loading of protein extracts*. (***C**) Immunoblot analysis of HEK cells expressing wild-type or mutant NKCC2 proteins, as indicated, using anti-Myc antibodies. (**D**,**E**) Quantitative analysis of NKCC2 protein abundance in OKP cells. Data were normalized with the loading control (Ponceau staining) from the same lane in each membrane and are expressed as percentage of control (WT NKCC2) *±* SE (**D**). The maturation efficiency of NKCC2 was estimated as the ratio of the mature vs. immature form of the cotransporter in OKP cells (**E**). Circles, squares and triangles represent individual data points for each tested condition (WT NKCC2, DAALA, AAALE and AAALA) as indicated. WT NKCC2, n = 6. DAALA, n = 3. AAALE, n = 5. AAALA, n = 4. ^#^ *p* < 0.05; * *p* < 0.0001 versus WT NKCC2. (**F**) Quantitative analysis of NKCC2 maturation efficiency in HEK cells. Data are expressed as percentage of control *±* SE. * *p* < 0.0001 (n = 3) versus WT NKCC2.

We previously demonstrated that the BS1 mutation Y998X, which deletes the last 101 amino acids of NKCC2 COOH terminus, causes the retention of the cotransporter at the ER [8]. Within this distal region of NKCC2 COOH-terminus, we identified several di-leucine-like motifs that are required for the ER exit of the cotransporter [8,9]. However, whether these motifs are sufficient for anterograde trafficking of NKCC2 remains to be determined. Moreover, while these hydrophobic motifs may serve as ER export signals [24], they may also simply provide a docking site for machinery proteins implicated in protein proper folding to a state competent for transport out of the ER [25]. Hence, we sought to further closely analyze the sequence of the last 101 amino acids of the NKCC2 COOH-terminus to check whether it contains a pure ER export signal. Among the motifs identified as ER pure export signals in ion channels and transporters are the di-acidic D/E-X-D/E motifs [18,19]. Interestingly, sequence analysis of the NKCC2 C-terminus revealed the presence of ^1019^DAELE^1023^ motif located downstream from BS1 mutation Y998X (Figure 1A). To explore the potential role of these residues in the export of NKCC2 from the ER, we generated a series of mutants in which D and/or E residues were substituted by alanine in the context of full-length NKCC2. In agreement with our previous findings, exogenously expressed NKCC2 in renal cultured cells was detected in two major forms: the complex *N*-glycosylated and mature form around 170 kDa, and the core/high-mannose immature form around 130 kDa [8,9,26]. As can be seen in Figure 1B,D,E, mutating ^1021^ELE^1023^ (^1021^EXE^1023^) of ^1019^DAELE^1023^ motif to ^1021^ALA^1023^ (DAALA) slightly decreased the abundance of the complex-glycosylated form of NKCC2 and the maturation efficiency (ratio of the mature vs. immature form of NKCC2) of the cotransporter. Interestingly, mutation of ^1019^DAE^1021^ (^1019^DXE^1021^) of the same motif to ^1019^AAA^1021^ (AAALE) had a much greater effect on the cotransporter, given that it massively reduced not only the expression of the fully glycosylated and mature form of NKCC2 (Figure 1B, *lanes 3, 6* and *9*) but also, in particular, its maturation efficiency (Figure 1D,E). These data strongly suggest that the ^1019^DAE^1021^ motif, alone, plays a crucial role in NKCC2 maturation. Consequently, simultaneous mutations of D, E and E residues to generate ^1019^AAALA^1023^ mutant almost completely abolished the complex-glycosylation of NKCC2 (Figure 1B, *lanes 10* and *12*) and heavily impaired the maturation efficiency of the cotransporter (Figure 1B–E). Finally, because protein subcellular distributions and ER export machinery may depend on the expression system used, we sought to confirm our observations by conducting experiments in another cell line, i.e., HEK293 cells. As shown in Figure 1C (*lanes 2* and *3*) and 1F, ^1019^AAALE^1023^ and ^1019^AAALA^1023^ mutations strikingly decreased the maturation efficiency of NKCC2 in HEK cells by 64% and 80% respectively, reproducing the effects observed in OKP cells, clearly indicating that the ^1019^DAELE^1023^ signal plays a critical role in NKCC2 maturation independently of the expression system.

### 2.2. Specificity for the Requirement of ^1019^DAE^1021^ Motif in NKCC2 Maturation

**Figure 2:**
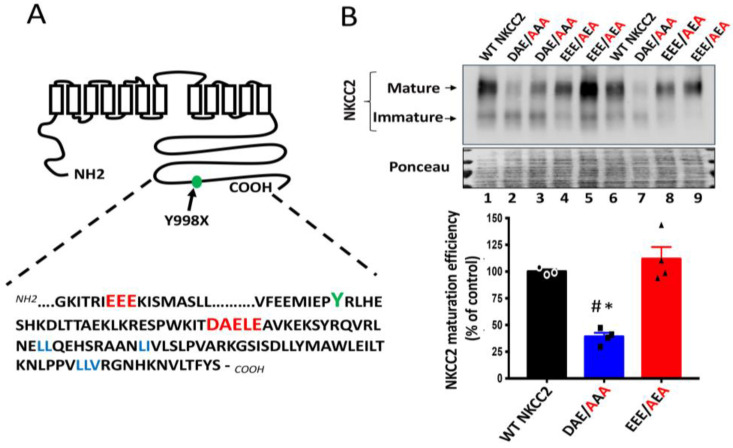
Comparison between the roles of ^949^EEE^951^ and ^1019^DAE^1023^ motifs in NKCC2 processing. (**A**) The distal region of NKCC2 C-terminal tail harbors two consensus di-acidic (D/E-X-D/E) motifs (red), ^949^EEE^951^ and ^1019^DAELE^1023^, located upstream and downstream from Y^998^ residue (green), respectively. (**B**) Immunoblot analysis of OKP cells transiently transfected with wild-type or mutated NKCC2 proteins that were generated by mutating the ^1019^DAE^1023^ motif to AAA (DAE/AAA) or mutating the ^949^EEE^951^ motif to AEA (EEE/AEA). Forty-eight hours post-transfection, total cell lysates were separated by SDS-PAGE and Myc-tagged NKCC2 proteins were revealed by anti-Myc antibodies. Lower panel, Densitometric analysis of the maturation efficiency of WT NKCC2 or mutant proteins, estimated as the ratio of the mature vs. immature form of the cotransporter. Data are expressed as percentage of control (WT NKCC2) ± S.E. WT NKCC2, n = 3. DAE/AAA, n = 4. EEE/AEA, n = 4. ^#^ *p* < 0.002 versus WT NKCC2. * *p* < 0.0004 vs. EEE/AEA.

The potential significance of the above findings is that, in addition to the previously identified di-leucine like motifs, the ^1019^DAELE^1023^ residues, in particular the ^1019^DAE^1021^ motif found downstream BS1 from Y998X mutation in NKCC2 COOH terminus, could be very important sorting determinants for trafficking to the plasma membrane of TAL cells. Thus, naturally occurring mutations interfering with ^1019^DAELE^1023^ residues, in particular with the ^1019^DAE^1021^ motif, could lead to BS type 1. Interestingly, further sequence analysis of NKCC2 COOH-terminus revealed also the presence of another di-acidic D/E-X-D/E signal, which is ^949^EEE^951^*,* but this motif is located upstream of BS1 mutation Y998X (Figure 2A). This prompted us to compare the effect of mutating ^1019^DAE^1021^ (^1019^DXE^1021^) motif with that of the ^949^EEE^951^ (^949^EXE^951^) motif, located downstream and upstream of Y998X, respectively, on NKCC2 maturation. Interestingly, as can be seen in Figure 2B, whereas mutating ^1019^DAE^1021^ to AAA (DAE/AAA) greatly disrupted complex-glycosylation and maturation efficiency of NKCC2, mutation of the ^949^EEE^951^ motif to AEA (EEE/AEA) had no effect on the cotransporter expression and maturation, illustrating the specificity of the ^1019^DAE^1021^ implication in the ER exit of the cotransporter. Hence, these results further highlight the importance of the region downstream of BS1 mutation Y998X in NKCC2 trafficking. Moreover, these data provide additional evidence for the importance and specificity of the ^1019^DAE^1021^ motif in NKCC2 exit from the ER and its relevance to BS1.

### 2.3. Effect of ^1019^AAALE^1023^ and ^1019^AAALA^1023^ Mutations on NKCC2 Surface Expression

**Figure 3:**
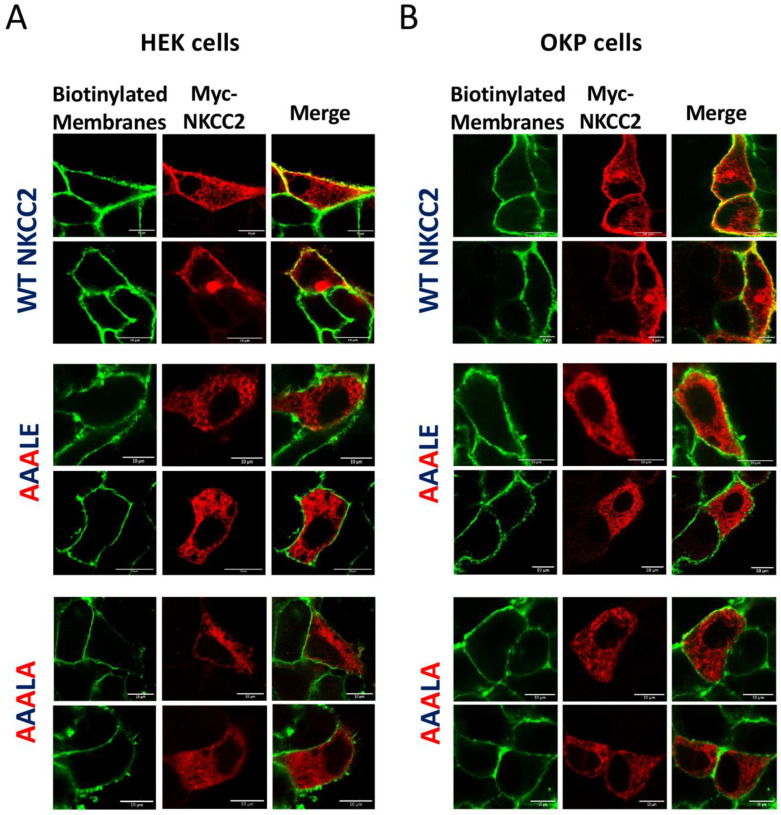
Mutating the ^1019^DAELE^1023^ motif impairs NKCC2 cell surface expression. Membrane proteins of confluent HEK (**A**) or OKP cells (**B**) transiently transfected with wild-type NKCC2 or ^1019^AAALE^1023 1019^AAALA^1023^ mutants, were biotinylated at 4 °C with the biotinylation reagent sulfo-NHS-SS-biotin. Then, the monolayers were fixed and stained for Myc-tagged NKCC2 proteins (Texas-red, red) and cell surface biotin (avidin-Cy2, green). The stained specimens were evaluated by confocal microscopy. The yellow color in the merged image indicates co-localization of myc-NKCC2 (red) with biotin-Cy2 (green). Bars, 10 μm.

We previously provided evidence that only the complex-glycosylated and mature form of NKCC2 is delivered to the cell surface [8,9,26]. Given the very low expression and/or absence of mature NKCC2 forms in cells expressing ^1019^AAALE^1023^ or ^1019^AAALA^1023^ mutants, one would anticipate that the delivery to the cell surface of these mutants is impaired. Consequently, to elucidate the consequence of ^1019^AAALE^1023^ and ^1019^AAALA^1023^ on NKCC2 processing, we next investigated their effects on NKCC2 expression at the plasma membrane. Toward that, we performed cell surface biotinylation to compare the subcellular localization of wild-type NKCC2 and the mutant proteins by immunostaining. As expected, immunocytochemistry analysis revealed that the staining of WT NKCC2 proteins co-localized with biotinylated cell surface proteins (Figure 3A), indicating correct targeting of the cotransporter to the cell surface. In contrast, HEK cells transfected with ^1019^AAALE^1023^ or ^1019^AAALA^1023^ mutants displayed an immunofluorescence staining pattern that was more restricted to a perinuclear ER-like distribution. Indeed, these cells did not show any noticeable colocalization with biotinylated cell-surface proteins, indicating that these mutants were not expressed at the cell surface (Figure 3A). Again, the effects of ^1019^AAALE^1023^ and ^1019^AAALA^1023^ mutations on NKCC2 were independent of the expression system, given that similar results were obtained in OKP cells (Figure 3B). On the basis of these data, we concluded that the ^1019^DAELE^1023^ motif is required for normal and efficient forward trafficking of mature proteins to the plasma membrane.

### 2.4. Localization of ^1019^AAALE^1023^ and ^1019^AAALA^1023^ Mutants in the ER

**Figure 4:**
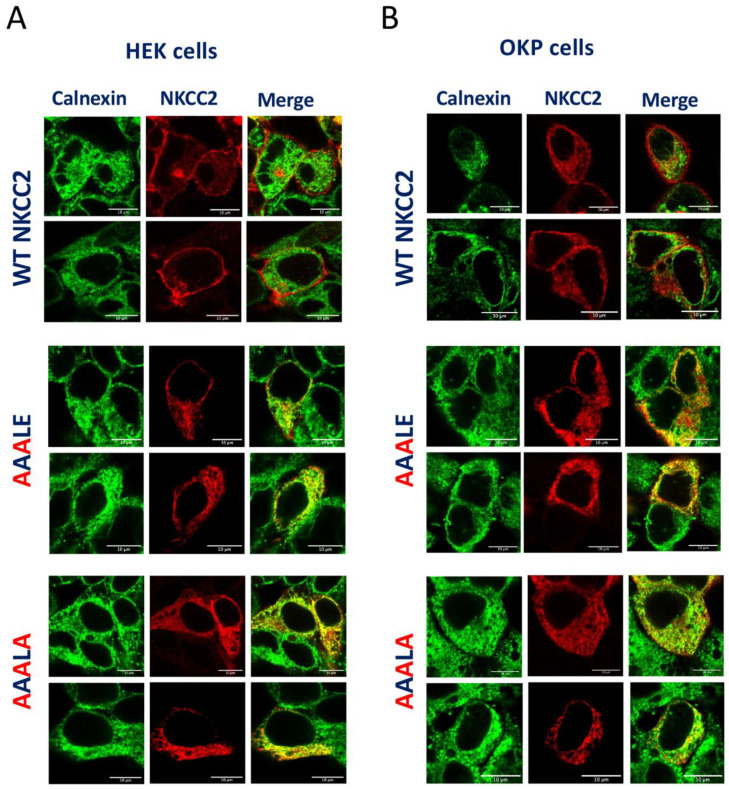
^1019^AAALE^1023^ and ^1019^AAALA^1023^ are not expressed at the cell surface due to retention in the endoplasmic reticulum. Transiently transfected HEK cells (**A**) or OKP cells (**B**) with wild-type or ^1019^AAALE^1023^ and ^1019^AAALA^1023^, as indicated, were fixed and permeabilized, and then cells were stained with mouse anti-Myc (Texas red; red)) and rabbit anti-calnexin (fluorescein isothiocyanate; green). Yellow, overlap between the Myc tag of NKCC2 protein (red) and the ER marker (green), representing co-localization of the proteins at the ER. Bars, 10 μm.

The above data suggest that mutating the D, E and E residues of ^1019^DAELE^1023^ motif results in defective processing of NKCC2 from the ER to the Golgi apparatus, and consequently in lower expression or absence of complex-glycosylated and mature form of the cotransporter protein at the cell surface. Based on these findings, one would expect that the corresponding mutants would be retained either partially or totally in the ER. To test this hypothesis, we analyzed the subcellular localization of WT NKCC2 and its mutants with ER marker calnexin, using immunofluorescence confocal microscopy. As can be seen in Figure 4A,B in HEK cells and OKP cells, respectively, WT NKCC2 displayed, again as expected, strong plasma membrane expression. Notably, despite some staining patterns surrounding the calnexin staining, the ^1019^AAALE^1023^ mutant was largely trapped in the ER as illustrated by its major colocalization with the ER marker. Even more impressive, the triple mutant ^1019^AAALA^1023^ completely accumulated in a perinuclear compartment as judged by its extensive colocalization with the calnexin. In sum, these data clearly show that ^1019^AAALE^1023^ and ^1019^AAALA^1023^ mutants are largely retained in the ER, indicating that the ^1019^DAELE^1023^ motif is required to transport NKCC2 from the ER to the cell surface. Of note, to further confirm that ^1019^AAALA^1023^ proteins were trapped in the ER, we evaluated its sensitivity to endoglycosidase-H (Endo-H). Endoglycosidase-H cleaves *N*-linked oligosaccharides on high mannose and hybrids (but not complex-type) oligosaccharides making it useful for examining the maturation of NKCC2 proteins as they move through the ER-Golgi compartments. In this regard, we previously demonstrated that, in contrast to the complex-glycosylated 170 kDa band NKCC2, the core glycosylated 130 kDa band and immature form of the cotransporter, is sensitive to Endo-H [8,9]. Accordingly, similar to the core-glycosylated and ER resident form of wild-type NKCC2 [8,9], ^1019^AAALA^1023^ mutant was also sensitive to endo-H, indicating that it was prevented from advancing beyond the ER to acquire endo-H resistance. In sum, this combination of biochemical and immunocytochemical experiments provides strong evidence that in the absence of a functional ^1019^DAELE^1023^ motif, NKCC2 failed to exit the ER.

### 2.5. Mutation of the ^1019^DAELE^1023^ Motif Impaired Maturation of the Cotransporter

**Figure 5:**
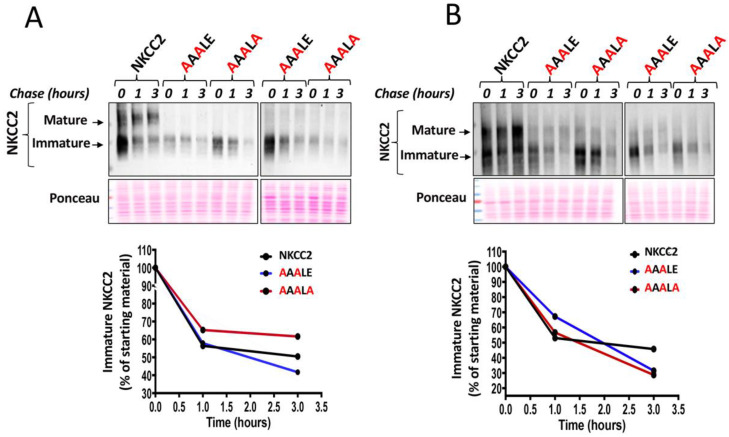
Mutation of the ^1019^DAELE^1023^ signal impairs NKCC2 maturation. (**A**,**B**), cycloheximide chase analysis of wild-type and mutated NKCC2 proteins. 14–16 h post-transfection, OKP cells (**A**) or HEK cells (**B**) transiently expressing WT NKCC2 or mutant proteins. ^1019^AAALE^1023^ or ^1019^AAALA^1023^, were chased for the indicated time after addition of cycloheximide. Total cell lysates were separated by SDS-PAGE and probed by anti-Myc antibodies. Lower panels**,** Quantitative analysis of the immature NKCC2 protein abundance. The density of the immature form of NKCC2 proteins was normalized to the density at time 0. Each point represents mean from two independent experiments.

To determine if the ER localization of NKCC2 mutants reflected a defect in maturation and/or enhanced degradation, we analyzed the role of the ^1019^DAELE^1023^ motif in the stability and biosynthetic processing of NKCC2 by a cycloheximide assay as described in our previous reports [7,9,27,28,29]. To that end, 14 to 16 h after transfecting OKP cells with Myc-tagged wild type or mutant NKCC2, cells were incubated with cycloheximide for 0, 1 and 3 h to block protein synthesis. During the chase period, NKCC2 levels were monitored by Western blot. As can be seen in Figure 5, wild-type NKCC2 protein was initially synthesized as the core-glycosylated and immature form before being gradually converted to a more slowly migrating band corresponding to the mature and complex glycosylated form of the cotransporter, the only form capable of reaching the cell surface. In kinetic analysis, the immature form of wild-type NKCC2 protein showed a progressive decrease with an estimated half-life of 3 h. Of note, the decrease represents the conversion of the immature to the mature form, as well as the degradation of the immature form of the NKCC2 protein. In contrast to WT NKCC2, the conversion from the immature form of 130-kDa to the mature form of NKCC2 was barely detectable during the chase period in OKP cells (Figure 5A) and HEK cells (Figure 5B) expressing ^1019^AAALE^1023^ and ^1019^AAALA^1023^ mutants, suggesting that mutations of D, E and E residues block protein sorting from the ER. Importantly, extrapolation of the decay curve obtained from the cells expressing NKCC2 mutants did not reveal a noticeable difference in the estimated half-life of ^1019^AAALE^1023^ and ^1019^AAALA^1023^ proteins when compared to WT NKCC2 (Figure 5A,B lower panels), suggesting that the absence of the terminally glycosylated form of the mutant proteins was not due to increased rates of degradation of mutant co-transporters, but was instead caused by defects in maturation. Altogether, these data demonstrate that ^1019^AAALE^1023^ and ^1019^AAALA^1023^ mutants clearly disrupt transport ER-to-Golgi trafficking of NKCC2, thereby impairing the cell surface expression of the cotransporter.

### 2.6. Functional Sar1/COPII Machinery Is Required for ER NKCC2 Export

**Figure 6:**
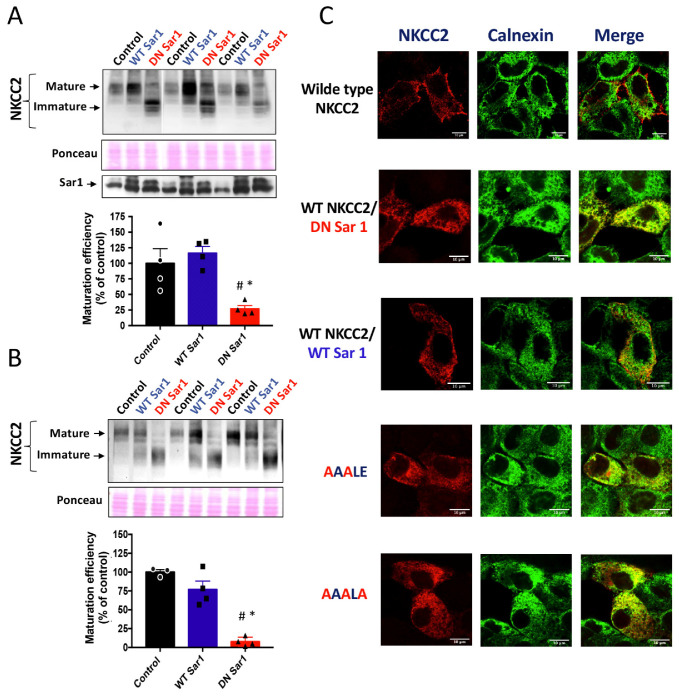
Loss of functional Sar1 GTPase inhibits ER NKCC2 export. (**A**,**B**) representative immunoblot showing the effect of overexpressing WT Sar1 or its dominant negative (DN Sar1) Sar1 ^H79G^ on NKCC2 processing. (**A**) HEK cells were transfected with NKCC2 alone or with WT Sar1 or DN Sar1. 48 h post-transfection, total cell extract from each sample was run on a parallel SDS gel and immunoblotted for total NKCC2 using Myc antibody. Middle panel, the same membrane stained with Ponceau S to illustrate the equal loading of protein extracts. Lower panel, Representative immunoblot illustrating the expression of endogenous Sar1 (control) and transfected WT Sar1 and its dominant negative in HEK cells. Sar1 proteins were detected using anti-Sar1A antibodies. (**B**) Quantitative analysis of the maturation efficiency of NKCC2 in the presence or absence of WT Sar1 or DN Sar1 in HEK cells, estimated as the ratio of the mature vs. immature form of the cotransporter. Data are expressed as percentage of control ± S.E. NKCC2 alone n = 3. NKCC2 with WT Sar1, n = 4. NKCC2 with DN Sar1, n = 4. ^#^ *p* < 0.0001 versus NKCC2 alone. * *p* < 0.0005 versus WT Sar1. (**C**) Representative immunoblot illustrating the effect of DN Sar1 in OKP cells. Lower panel, Quantitative analysis of the maturation efficiency of NKCC2 with or without WT Sar1 or DN Sar1 in OKP cells. Each point represents mean ± S.E of four independent experiments. ^#^ *p* < 0.02 versus NKCC2 alone. * *p* < 0.007 versus WT Sar1. (**C**) Comparison between the effects of mutating the ^1019^DAELE^1023^ motif and DN Sar1 on NKCC2 subcellular distribution. HEK cells transfected with mutated Myc-NKCC2 proteins as indicated, or with WT NKCC2 alone in the presence or absence of WT Sar1 or DN Sar1, were stained with mouse anti-Myc (Texas Red; red) and rabbit anti-calnexin (FITC; green). The yellow color indicates overlap between the Myc tag of NKCC2 protein (red) and the ER marker (green), representing co-localization of the proteins. Bars, 10 μm.

To properly exit the ER, membrane proteins bearing the ER export signal interact, in general, specifically with the COPII-coat machinery [24]. The COPII-coat machinery is composed of the small GTPase Sar1 and the heteromeric protein complexes Sec23–Sec24 and Sec31–Sec13 [20,24]. By mediating the GDP/GTP exchange reaction, Sar1 GTPase initiates the assembly of cargo into COPII vesicles, which mediate the sorting of newly synthesized proteins from the ER to the ER-Golgi intermediate compartment [20,24]. Hence, COPII vesicle-mediated ER exit of cargo can be prevented with dominant negative mutants of Sar1 such as Sar1^H79G^, which is unable to hydrolyze GTP [18,30]. Consequently, to uncover whether the Sar1/COPII machinery participates in NKCC2 sorting, we compared the effect of co-expressing Sar1^H79G^ with that of WT Sar1 on NKCC2 expression and maturation. To that end, HEK cells and OKP cells were transiently transfected with Myc-NKCC2 plasmid either singly or in combination with Sar1 wild-type or Sar1 ^H79G^. As seen in Figure 6A, when WT SAR1 was co-expressed with NKCC2, the latter processed normally to the mature form as it does when it is expressed alone in control cells. Importantly, similar to the effect of the ^1019^DAELE^1023^ motif (Figure 1), co-expression of Sar1^H79G^ heavily reduced the abundance NKCC2 complex-glycosylated form when compared to WT Sar1, and strikingly impaired its maturation efficiency (Figure 6A). Again, similar results were obtained in OKP cells (Figure 6B. Hence, our data clearly indicate that the Sar1^H79G^ mutant was capable of preventing NKCC2 exit from the ER. To elucidate the consequence of SAR1 ^H79G^ effect on NKCC2 maturation, we compared the effect of ^1019^AAALE^1023^ and ^1019^AAALA^1023^ mutants with that of Sar1 dominant negative on the subcellular distribution of the cotransporter by co-immunocolocalization using the ER marker, calnexin. As expected, when transfected alone, or in the presence of WT Sar1, NKCC2 staining at the cell periphery clearly surrounded the calnexin signal. By contrast, Sar1^H79G^ co-expression reproduced the effect of ^1019^AAALE^1023^ and ^1019^AAALA^1023^ mutants, by abolishing NKCC2 surface expression and generating an intensive perinuclear pattern of staining for NKCC2 with extensive co-localization with the ER marker calnexin (Figure 6C). Hence, these results strongly indicate the ER export of NKCC2 requires a functional Sar1 GTPase. Collectively, our data provide evidence that efficient export of NKCC2 from the ER and trafficking to the cell surface is dependent on the ^1019^DAELE^1023^ motif and a COPII-mediated mechanism.

### 2.7. D^1019^ and E^1021^ Residues of the ^1019^DAE^1021^ Motif Are Conserved in the COOH-Terminal Tails of All Na-Cl Co-Transporters

**Figure 7:**
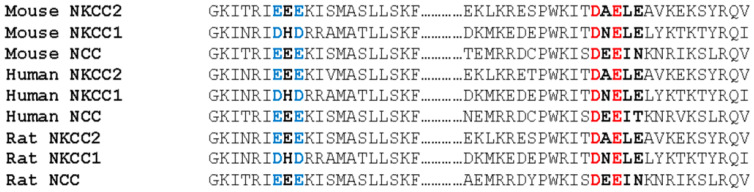
D^1019^ and E^1021^ of the ^1019^DAE^1021^ motif are conserved. Amino acid sequence alignment of the carboxyl termini of the structurally related Na-Cl cotransporters NKCC2, NKCC1 and NCC. The E^949^ and E^951^ residues of the ^949^EEE^951^ (^949^EXE^951^) motif are in blue. In red, the D^1019^ and E^1021^ residues of the ^1019^DAE^1021^ (^1019^DXE^1021^) motif. See “Results” for additional details.

The data described above clearly show that the ^1019^DAELE^1023^ signal and, in particular, the D^1019^ and E^1021^ residues of ^1019^DAE^1021^ (^1019^DXE^1021^) motif, play a crucial role in ER-to-Golgi trafficking and efficient transport to the cell surface of NKCC2. Given that amino acid residues that play important roles in protein function are often conserved, we sought to examine whether the D^1019^ and E^1021^ residues of ^1019^DXE^1021^ were conserved among the Na-Cl cotransporter family members NKCC1, NKCC2 and NCC. Interestingly, in contrast to the A^1020^ residue, the results revealed that D^1019^ and E^1021^ are fully conserved in the COOH-terminal tails of all structurally related members of the Na-Cl cotransporters family (Figure 7, in red). Importantly, D^1019^ and E^1021^ residues were conserved not only between NKCC1, NKCC2 and NCC, but also between species (mouse, human and rat). Of note, each E residue of the ^949^EEE^951^ (^949^EXE^951^) motif (Figure 7, in blue) was conserved in the apically located Na-Cl cotransporter NCC, and was replaced by another acidic amino acid (D) in the basolateral Na-K-2Cl cotransporter NKCC1. Although our results provide evidence that the ^949^EXE^951^ motif is not required for NKCC2 exit from the ER (Figure 2), one cannot completely discount the possible implication of this motif in the correct sorting and vectorial delivery the Na-Cl cotransporter proteins in epithelial cells. Taken together, these data lend further support to the role of ^1019^DAE^1021^ motif in ER export of NKCC2 and strongly suggest that it may function as a common ER export signal for all members of Na-Cl cotransporters family.

## 3. Discussion

We previously showed that export from the ER represents the limiting step in NKCC2 trafficking to the cell surface [7,8,9,27,28,29,31]. Herein, we reveal the requirement of the COPII budding machinery together with di-acidic motifs, ^1019^DAELE^1023^, for NKCC2 export from the ER. Mutation of ^1019^DAELE^1023^ to ^1019^AAALA^1023^ caused the retention of the cotransporter in the ER and thereby impaired its trafficking to the cell surface. Interestingly, the sequence analysis of NKCC2 COOH-terminus also revealed another di-acidic motif, ^949^EEE^951^, but the latter had no effect on NKCC2 processing, proving the existence of specific molecular components and signals regulating ER-to Golgi trafficking of NKCC2.

Several cytosolic ER export signals, such as adjacent bulky hydrophobic motifs (FF, FY, LL, IL, or YYM), di-acidic motifs ((D/E)*X*(D/E)), dibasic motifs ((R/K)*X*(R/K)), a combination of motifs, or multiple cooperating signals, have been described within the terminal COOH tail of different transmembrane proteins [14,15,19,32]. Moreover, several studies have demonstrated that COOH-terminal mutations are responsible for several genetic disorders [33,34,35]. Consistent with this, we previously showed that BS1 mutations Y998X, D918fs and N984fs, interfering with three di-leucine like motifs in COOH terminus, cause defective ER exit of the cotransporter. Here, we report that within the same distal region NKCC2 C-terminus, we identified ^1019^DAELE^1023^ as a new signal required for NKCC2 ER export and efficient delivery to the plasma membrane, showing that the previously described hydrophobic motifs are essential, but not sufficient, for proper intracellular trafficking of the cotransporter. It is important to notice that the ^1019^DAELE^1023^ signal include the ^1019^DAE^1021^ (^1019^DXE^1021^) and the ^1021^ELE^1023^ (^1021^EXE^1023^) sequence, and each one resembles the di-acidic (D/E)X(D/E) ER exit motif. However, our results revealed that mutating ^1021^ELE^1023^ had only a slight effect on NKCC2 maturation, whereas the mutation of the ^1019^DAE^1021^ motif heavily affected the ER exit and cell surface expression of the cotransporter. Interestingly, triple mutation D, E and E residues of ^1019^DAELE^1023^ to alanines (^1019^AAALA^1023^) completely prevented NKCC2 maturation, clearly indicating the requirement of the whole ^1019^DAELE^1023^ motif in NKCC2 ER export. In support of these findings, a cycloheximide chase assay and immunocytochemistry experiments showed that in the absence of a functional ^1019^DAELE^1023^ motif, NKCC2 proteins were prevented from exiting the ER, lost their ability to convert to the mature of NKCC2 and failed to reach the plasma membrane. It is worth emphasizing that the di-acidic motifs are considered, in general, to be pure ER export signals [18,19], whereas the di-leucine-like and hydrophobic motifs can also be involved in proper protein folding. In support of this notion, Nezu et al. [25] demonstrated that mutating a highly conserved hydrophobic tetrad ILLV motif in the basolateral isoform of Na-K-2Cl cotransporter NKCC1, which contains the ^1081^LLV^1083^ motif required for NKCC2 exit from the ER, leads to the misfolding and concomitant aggregation of the cotransporter. Consequently, it is conceivable that the mechanism of NKCC2 ER retention caused by ^1019^DAELE^1023^ mutations is different from that observed following the mutation of di-leucine like motifs. Indeed, it is very likely that ^1019^AAALE^1023^ and ^1019^AAALA^1023^ mutants (in particular ^1019^AAALA^1023^) have much less profound conformational abnormalities. Generally, membrane proteins carrying di-acidic motifs congregate at COPII-enriched ER exit sites [24]. Accordingly, when the dominant negative of Sar1 GTPase, the initiator of COPII-complex machinery, was overexpressed in our cells, the ER exit and surface expression of NKCC2 were impaired, indicating that ER export of the cotransporter is COP-II-dependent. In sum, together with our previous reports, our data provide evidence that the ER exit of NKCC2 is orchestrated by multiple sorting signals, including di-leucine-like motifs and di-acidic export codes. All these different sorting signals may act independently or in coordination to govern NKCC2 maturation and cell surface expression. Obviously, further extensive studies elucidating interaction and network dynamics within NKCC2 C-terminus are needed to provide more insights toward understanding the role of each sorting signal in the control of the cotransporter trafficking.

Many genetic diseases have been linked to mutations affecting the sorting of various proteins, in particular those belonging to the groups of transporters and channels [36,37,38]. These genetic defects can directly interfere with protein transport by affecting conventional trafficking at any step during the journey to the plasma membrane [36]. Among the mechanisms involved, ER retention and ERAD constitute the most common cause of a wide range of disorders called conformational diseases [38,39,40]. For instance, mutations in the genes encoding ROMK (renal outer-medullary K+ channel) cause type II Bartter syndrome because ROMK mutant is retained in the ER and is unable to reach the apical membrane of TAL cells [41]. In other cases, diseases associated with defective protein trafficking were triggered by mutations affecting internalization and sorting of plasma membrane proteins, such as mutations within an endocytosis motif in the gene encoding epithelial Na^+^ channel (ENaC), altering ENaC abundance at the plasma membrane and consequently causing Liddle’s syndrome [42,43]. Mistargeting mutations in epithelial cells lead also to defective polarized trafficking to the apical or basolateral membranes, causing rerouting of the mutant protein to the incorrect membrane domain, as, for example, in distal renal tubular acidosis caused by mutations in the chloride-bicarbonate exchanger AE1 and aquaporin 2 disrupting their basolateral and apical targeting signals, respectively [38,44,45]. Last but not least, a disease can also be caused by mutations that interfere with ER export signals. In support of this possibility, Taneja et al. reported that the surface expression of K(ATP) channels is critically dependent on the COPII machinery, and any mutation that causes the abrogation of the di-acidic ER exit signal of the channel leads to congenital hyperinsulinism [46]. Likewise, based on the data in the present work, we propose that any naturally occurring NKCC2 mutation interfering with the ^1019^DAELE^1023^ export signal, and in particular with the ^1019^DAE^1021^ motif, could lead to BS type 1. Together with our previous reports demonstrating that BS type 1 is among diseases linked to ER retention and ERAD pathways [7], our findings further highlight the importance of ER quality control and ER export in the control of NKCC2 routing to the cell surface in renal health and disease. With respect to this idea, we and others have previously reported that inactivating MAGE-D2, a protein that protects NKCC2 and NCC against ER associated degradation, leads to BS type 5, which is the most severe form of antenatal Bartter syndrome described so far, as judged by both onset and severity of polyhydramnios and the high rate of mortality observed in patient carriers of MAGE-D2 mutations [28,47,48].

**Figure 8:**
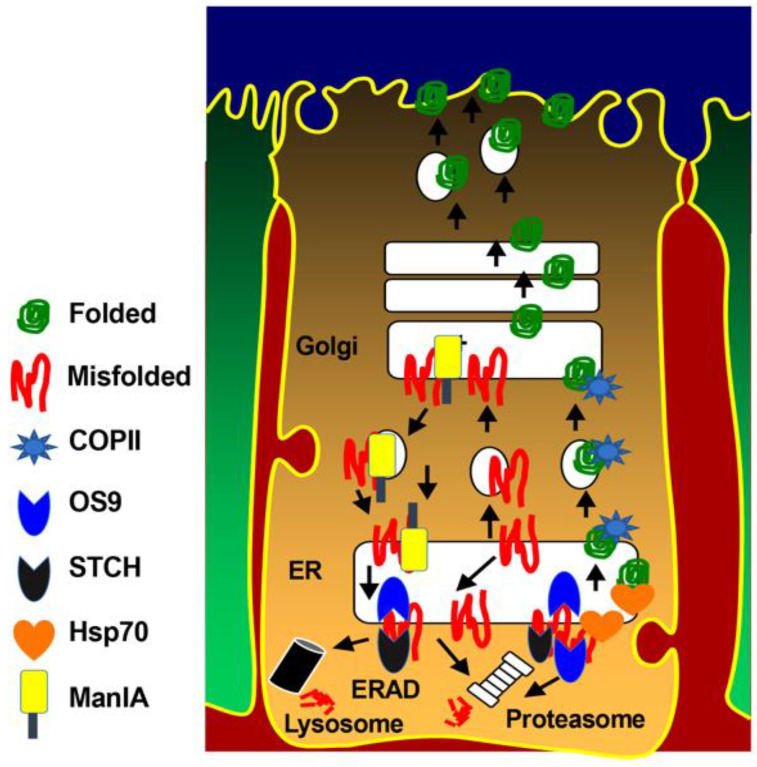
Molecular determinants of NKCC2 ER export and ER-associated degradation. Properly folded NKCC2 proteins use di-leucine like motifs and di-acidic codes to exit the ER through the COPII budding machinery. OS9 mediates the retention of misfolded NKCC2 proteins at the ER and their ERAD by the proteasome [27]. STCH role in the ERAD of NKCC2 involves both the proteasome and lysosome pathways [31]. Golgi ManIA promotes efficient ERAD of NKCC2 by capturing misfolded NKCC2 proteins that escaped ER quality control and delivers them back to the ER [29]. Finally, Hsp70 protects NKCC2 against ERAD and promote its maturation [31].

In conclusion, our current findings indicate that efficient anterograde trafficking of the NKCC2 protein is critically dependent on the COPII budding machinery and the presence of functional di-leucine-like motifs and di-acidic ER export codes in the distal region co-transporter C-terminus. It is noteworthy that besides COPII, we previously identified several NKCC2 binding-proteins and ERAD components, such as OS9, STCH, Hsp70, and Golgi-Mannosidase IA (ManIA), that are involved in NKCC2 biogenesis [27,29,31]. Consequently, we propose here a model (Figure 8) whereby: (1) di-leucine-like motifs and di-acidic codes mediate the ER export of properly folded NKCC2 proteins through the COPII budding machinery; (2) OS9, STCH and Golgi ManIA promote efficient ERAD of misfolded NKCC2 proteins; (3) similar to MAGE-D2, Hsp70 protects the cotransporter against ERAD and promotes NKCC2 maturation. Obviously, further experiments are required to elucidate the precise molecular mechanisms behind each step of this model. Deciphering the molecular basis of aberrant trafficking and processing of NKCC2 and its related disorders could be a powerful tool to reveal completely new and unanticipated signals and avenues in the regulation of not only NKCC2 but also of the structurally related ion transporters such the kidney specific Na-Cl cotransporter NCC and the ubiquitous Na-K-2Cl cotransporter NKCC1. Indeed, the expansion of pharmaceutical compounds that are being generated thanks to the increasing knowledge of disease-related mechanisms modulating protein sorting may improve current treatments or, perhaps, provide novel therapeutic strategies for incurable related disorders.

## 4. Materials and Methods

### 4.1. Plasmid Constructions and Site Directed Mutagenesis

The cDNA encoding mouse NKCC2 was fused at the C-terminal end of Myc (Myc- NKCC2) using pCMV-Myc vectors as previously reported [26]. The pcDNA3 plasmids encoding wild-type and dominant negative Sar1 GTPase [30] were a kind gift from Dr. Guangyu Wu (Louisiana State University, LA, USA). Double, and triple mutations of D^1019^, E^1021^ and L^1023^ residues of the ^1019^DAELE^1023^ motif to alanine(s) were performed within the context of full-length WT NKCC2 using pCMV-Myc -NKCC2 construct and according to the QuikChange protocol (QuikChange site-directed mutagenesis kit; Stratagene). All mutations were confirmed by sequencing.

### 4.2. Cell Culture

Opossum kidney cells (OKP cells) were maintained in DMEM (Gibco 42430) supplemented with 10% fetal bovine serum (Eurobio, Les Ulis, France), penicillin (100 U/mL), and streptomycin (100 U/mL) at 37 °C in a humidified atmosphere containing 5% CO_2_. Human embryonic kidney 293 cells (HEK) were grown in DMEM media complemented with 10% fetal bovine serum and 1% penicillin/streptomycin. For plasmid DNA transfection, cells were grown to 60–70% confluence before being transiently transfected for 5 h using Lipofectamine plus kit according to manufacturer’s instructions (Invitrogen, Paris, France).

### 4.3. Protein Preparation and Immunoblotting

After transfection, cells were washed with cold PBS before being solubilized in a lysis buffer containing 120 mM Tris/Hepes, pH 7,4; 150 mM NaCl, 5 mM EDTA, 3 mM KCl; 1% (*v/v*) Triton X-100 and protease inhibitors (Complete Roche 1697498, Meylan, France). Samples were then harvested and centrifuged at 16,000 rpm for 15 min at 4 °C. Protein expression levels were assessed after normalizing and loading equal amounts of total protein for 7.5% SDS-PAGE separation and immunoblotting with the antibodies of interest and horseradish peroxidase-conjugated secondary antibody. The primary antibodies used in this study were mouse anti–Myc (Takara, Clontech, Saint-Germain-en-Laye, France) and anti-Sar1A (Invitrogen). Proteins were visualized by enhanced chemiluminescence detection (Thermo Fisher Scientific, Les Ulis, France) according to the manufacturer′s instructions.

### 4.4. Immunocytochemistry

Twenty-four to forty-eight h post-transfection, confluent cells were washed with PBS^++^ (pH 8, 1 mM MgCl_2_, and 0.1 mM CaCl_2_). Cells were then fixed with 2% paraformaldehyde in PBS for 20 min at room temperature before being incubated with 50 mM NH_4_Cl, permeabilized with 0.1% Triton X-100 for 1 min. To block nonspecific antibody binding, cells were incubated with DAKO (antibody diluent with background-reducing components) for 30 min. Fixed cells were incubated for 1h at room temperature with the primary antibody of interest. To assess protein expression at the cell surface, confluent cells were first incubated at 4 °C for 1 h in PBS^2+^ containing 1 mg/mL NHS-biotin. Cells were rinsed three times in rinsing solution with 100 mm glycine and reincubated at 4 °C in the same solution for 10 min. After washing with PBS^2+^, the monolayers were fixed and stained for cell surface biotin (avidin-Cy2; green). Mouse anti-Myc were visualized with Texas Red-coupled secondary antibodies. Rabbit anti-calnexin was visualized with FITC-coupled IgG antibodies. Cells were then washed with PBS and mounted with Vectashield. The primary antibodies used in this study were the following: mouse anti–Myc (Takara, Clontech, Saint-Germain-en-Laye, France), rabbit anti-Calnexin (Abcam). The secondary antibodies used were the following: goat anti-rabbit FITC (DakoCytomation, Trappes, France), goat anti-mouse Texas red (Invitrogen), Alexa Texas Red conjugated anti-mouse (Jackson ImmunoResearch, Ely, UK).

### 4.5. Cycloheximide-Chase Assays

To monitor the stability and maturation of NKCC2, cycloheximide was added at a concentration of 100 μM to OKP or HEK cells 14–16 h post-transfection with NKCC2 plasmids. For the chase period, cell lysates were collected at 0, 1, and 3 h after cycloheximide treatment and analyzed by immunoblotting.

### 4.6. Statistical Analyses

Results are expressed as mean ± SE. Differences between means were evaluated using paired or unpaired t test or ANOVA as appropriate. *p* < 0.05 was considered statistically significant.

## Data Availability

The data that support the findings of this study are available on request from the corresponding author.
